# Investigation of Donor-like State Distributions in Solution-Processed IZO Thin-Film Transistor through Photocurrent Analysis

**DOI:** 10.3390/nano13232986

**Published:** 2023-11-21

**Authors:** Dongwook Kim, Hyeonju Lee, Kadir Ejderha, Youngjun Yun, Jin-Hyuk Bae, Jaehoon Park

**Affiliations:** 1 School of Information Science, Hallym University, Chuncheon 24252, Republic of Korea; d.kim@hallym.ac.kr (D.K.); hjlee@hallym.ac.kr (H.L.); 2 Department of Physics, Faculty of Science and Arts, Bingol University, Bingöl 12000, Turkey; kejderha@bingol.edu.tr; 3 School of Semiconductor·Display Technology, Hallym University, Chuncheon 24252, Republic of Korea; youngjun.yun@hallym.ac.kr; 4 School of Electronics Engineering, Kyungpook National University, Daegu 41566, Republic of Korea; 5 School of Electronic and Electrical Engineering, Kyungpook National University, Daegu 41566, Republic of Korea; 6 Department of Electronic Engineering, Hallym University, Chuncheon 24252, Republic of Korea

**Keywords:** solution-processed IZO semiconductor, thin-film transistor, density of donor-like states, photo-excited carrier, photocurrent

## Abstract

The density of donor-like state distributions in solution-processed indium–zinc-oxide (IZO) thin-film transistors (TFTs) is thoroughly analyzed using photon energy irradiation. This study focuses on quantitatively calculating the distribution of density of states (DOS) in IZO semiconductors, with a specific emphasis on their variation with indium concentration. Two calculation methods, namely photoexcited charge collection spectroscopy (PECCS) and photocurrent-induced DOS spectroscopy (PIDS), are employed to estimate the density of the donor-like states. This dual approach not only ensures the accuracy of the findings but also provides a comprehensive perspective on the properties of semiconductors. The results reveal a consistent characteristic: the Recombination–Generation (R-G) center energy E_T_, a key aspect of the donor-like state, is acquired at approximately 3.26 eV, irrespective of the In concentration. This finding suggests that weak bonds and oxygen vacancies within the Zn-O bonding structure of IZO semiconductors act as the primary source of R-G centers, contributing to the donor-like state distribution. By highlighting this fundamental aspect of IZO semiconductors, this study enhances our understanding of their charge-transport mechanisms. Moreover, it offers valuable insight for addressing stability issues such as negative bias illumination stress, potentially leading to the improved performance and reliability of solution-processed IZO TFTs. The study contributes to the advancement of displays and technologies by presenting further innovations and applications for evaluating the fundamentals of semiconductors.

## 1. Introduction

Solution-processed indium–zinc-oxide thin-film transistors (IZO TFTs) have gained significant importance in the display market owing to their promising characteristics such as transparency, flexibility, and high electron mobility [1,2,3,4,5]. However, the widespread adoption of IZO TFTs has been hindered by a critical issue known as negative bias illumination stress (NBIS), which leads to device instability over time [6,7,8,9]. To comprehensively address this instability problem and enhance the performance and reliability of IZO TFTs, a thorough understanding of the charge transport mechanisms within an solution-processed oxide semiconductor is essential. One crucial aspect that requires attention is the distribution of donor-like states near the valence band edge. These states play a pivotal role in the overall charge transport within a device [10,11,12,13,14].

Because the electrical characteristics of the IZO TFT do not allow bipolar operation, it is difficult to obtain the distribution of donor-like states near the edge of the valence band, E_V_, from the gate bias [15,16,17]. That is, the surface energy band is bent upward by the negative bias, and even if holes accumulate in the donor-like states (even if the donor-like states are charged from neutral to positive), it does not significantly affect the overall current because there is insufficient energy to be elevated toward the conduction band, E_C_, level. Similarly, it is inefficient to measure the distribution of donor-like states below the Fermi energy level, E_F_, using thermal energy. In the case of the IZO semiconductor, holes excited from the donor-like states to the valence band by thermal energy do not affect the overall IZO current because the hole mobility is low. In this respect, photon energy is efficient in directly promoting electrons below the E_F_ energy level to the conduction band, E_C_. Electron-hole pairs (EHP) are consistently generated by photon energy at the recombination generation (R–G) center energy E_T_ and electrons are excited above the conduction band E_C_. These excited carriers prevail in the semiconductor and directly affect the current flow. The distribution of states (DOS) can be efficiently estimated by inversely calculating the excited carriers from the change in the photocurrent caused by light energy.

Studies analyzing the DOS distribution of oxide semiconductors using light have been conducted using various methods [18,19,20,21,22]. Several research groups have reported the threshold voltage shift and subthreshold voltage swing (S/S) slope of oxide TFT as a function of light intensity [23,24,25]. The donor-like states of oxide semiconductors have also been studied as a characteristic of photocurrent changes depending on the wavelength of light [26,27,28,29]. Moreover, studies have analyzed the transient current characteristics of oxide TFTs caused by light over time [30,31,32]. Particularly, Mativenga et al. analyzed the band-gap state change from the change in the photocurrent in terms of the thickness and wavelength of the semiconductor layer [33]. In this study, the light-induced change was attributed to the distribution of the donor-like states. This study adds that the distribution of the donor-like states is the cause of the NBIS.

Although numerous studies have suggested that the cause of IZO TFT photo-instability is the distribution of the donor-like states, methods for quantitatively calculating the DOS distribution directly in IZO TFTs have been relatively less studied. Among several computational methods, the photoexcited charge collection spectroscopy (PECCS) method efficiently presents the DOS distributions of ZnO semiconductors. In this approach, the threshold voltage of the ZnO TFT with respect to the wavelength of light is analyzed, and the band gap state distribution is calculated by differentiating the amount of change in the threshold voltage with the light energy [33,34,35]. This method of calculating the PECCS simply and efficiently presents the DOS distribution within the band gap, regardless of the semiconductor material. The PECCS calculation method is effective for analyzing the donor-like state distribution in the lower half of the semiconductor band gap; however, it has the following limitations when applied to the experiments in this study. The photocurrent characteristics varied not only with the change in wavelength but also with the measurement time. In the PECCS analysis, to extract the threshold voltage, it is necessary to measure the transfer curve in all scan wavelength bands. When this transfer curve is measured for every wavelength, the excited carriers accumulate in the TFT channel; therefore, the accumulation effect on the light energy is eventually added to the photocurrent characteristics [36]. Therefore, if the wavelength measurement interval is reduced, the total measurement time increases, and the amount of photocurrent in the TFT may also increase because of the decreased measurement interval.

In this study, the distribution of donor-like states in a solution-processed IZO semiconductor was analyzed with respect to the In concentration under irradiation conditions. In this approach, two calculation methods were used to analyze the photocurrent of the IZO TFT. First, the DOS was calculated quantitatively using PECCS. We then devised a photocurrent-induced DOS spectroscopy (PIDS) method to reduce the measurement time and interval. The donor-like state distribution through PIDS was validated by comparing the two calculation methods. In addition, the PIDS analysis was modified to develop the theoretical feasibility of the analysis results. Through this procedure, we approved the properties of photoexcited threshold voltage and photocurrent analysis and presented the detailed density of donor-like state distributions depending on the In molarity ratio.

## 2. Materials and Methods

**Figure 1:**
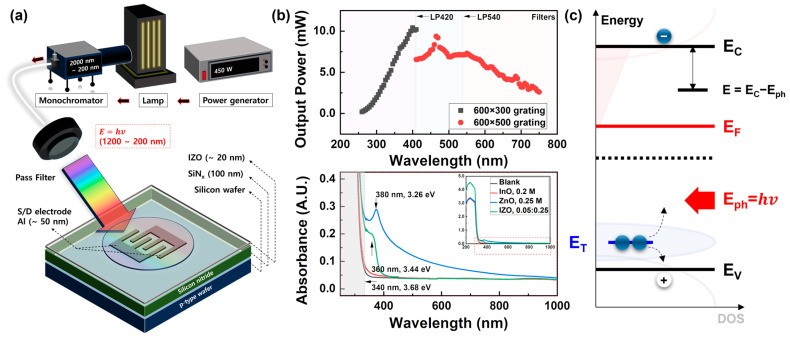
(**a**) Schematic illustration of the fabricated solution-processed IZO TFT and photocurrent measurement system. (**b**) Output power of photocurrent analysis system and UV/Vis. Light absorbance features of the solution-processed InO, ZnO, and IZO films. (**c**) Imaginary energy band diagram of the solution-processed IZO semiconductor and photo-excitation mechanism.

Solution-processed IZO TFTs were fabricated on p-type wafers with a 100 nm thick sputtered SiNx gate dielectric layer. For 20 nm thin IZO semiconductor layers, the indium nitrate hydrate (In_3_(NO_3_)_3_∙xH_2_O) (Sigma-Aldrich, St. Louis, MO, USA) and zinc nitrate hydrate (Zn_2_(NO_3_)_2_∙xH_2_O) (Sigma-Aldrich, St. Louis, MO, USA) were dissolved in 2-methoxyethanol (CH_3_O(CH_2_)_2_OH) (Sigma-Aldrich, St. Louis, MO, USA) solvent for more than 6 h. The specific molarity ratios for the In-doping effects are listed in Table 1. Subsequently, each solution was spin-coated onto a UV/O_2_ plasma-treated SiN_x_—Si substrate at 2000 rpm for 1 min. Soft baking was performed at 120 °C for solvent evaporation, followed by annealing at 550 °C, to complete the full oxidation of the IZO semiconductor. The 20 nm of Al source/drain electrodes deposited through thermal evaporation, with a width/length (W/L) ratio of 4000 μm/80 μm is shown in Figure 1a. The detailed fabrication processes and electrical characteristics can also be found in our previous studies [37].

Figure 1a shows a schematic of the photocurrent analysis system and provides an overview of the irradiation features used to characterize the electrical properties of IZO TFT. The light irradiation system (Dongwoo Optron, Gwangju-Si, Gyounggi-Do, Republic of Korea) comprised a power supply, a 450 W Xenon lamp, and a monochromator. This light source produces light wavelength within a range of 2000–200 nm, with practical usage; it is effectively delivered in the range of 1000–350 nm. The maximum output flux density measured using the TFT is approximately 0.1 mW cm^–2^. Figure 1b presents a detailed examination of the measured output power with the grating controls and pass filters employed to ensure uniform output power emission. Figure 1b also displays the ultraviolet/visible (UV/vis.) light absorbance of metal-oxide films, which are 0.5 M of ZnO, 0.2:0.25 M:M of IZO, and 0.4 M of InO. More detailed absorbance results, based on Table 1, are summarized in Supplementary Figures S1. Figure 1c shows the photon energy delivered through the light source to the IZO semiconductor within a band diagram.

All the electrical characteristics of the solution-processed IZO TFT were measured using a semiconductor analyzer (ELECS-420); (ELECS, Seoul, Republic of Korea) inside a dark box under a nitrogen atmosphere. This controlled chamber is necessary because UV-level light energy is susceptible to absorption by the humidity and oxygen in the air. The output power of the light source was measured using a handheld power meter (LaserCheck); (Coherent, Thebarton, SA, Australia), and the light absorbance was analyzed using UV/Vis. spectroscopy system (X-ma 3000 pc), (Human Co., Seoul, Republic of Korea).

## 3. Photo-Excited Charge Collection Spectroscopy

**Figure 2:**
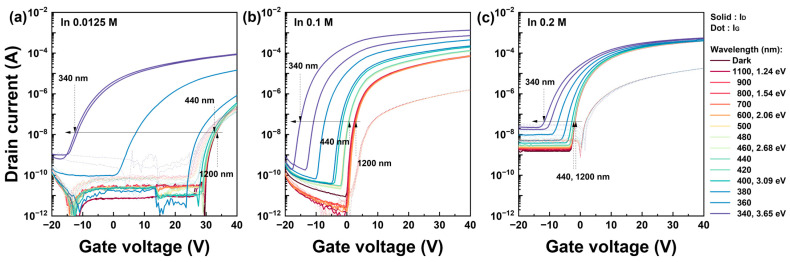
Photo-excited current characteristics in the transfer curve of solution-processed IZO TFTs. In the graph, the drain voltages of the TFT were 20 V, which represents the saturation condition. (**a**–**c**) indicate the transfer characteristic at different In molarity levels, while maintaining a constant Zn molarity 0.25 M: (**a**) 0.0125 M, (**b**) 0.1 M, and (**c**) 0.2 M, respectively.

The dependence of the photoexcited current characteristics on the photon energy was comprehensively evaluated using the PECCS analysis methods. Figure 2 shows the transfer characteristics of the solution-processed IZO TFTs as a function of the light wavelength. Irradiation was measured within a range of 1200–340 nm at 20 nm intervals, corresponding to photon energies of 1.24–3.65 eV. Furthermore, Figure 2 illustrates the behavior of the IZO TFTs at low, moderate, and high In molarity ratios, specifically 0.0125 M, 0.1 M and 0.2 M, respectively. More detailed electrical operation data concerning In molarity ratios are summarized in Supplementary Figure S2 and can be found in our previous study [37]. As shown in Figure 1, the currents remained consistent in the 1200–420 nm range regardless of the In concentration. However, within the 420–340 nm range, both the drain current and threshold voltage exhibited significant shifts with respect to the light wavelength. The maximum current shift was observed at 340 nm. Below 340–200 nm, the excited current gradually recovered to its initial state. We neglected these excited-state recoveries, speculating that they were caused by the monotonic decrease in the output power below 340 nm, as shown in Figure 1b. The most pronounced fluctuations in the on- and off-state currents, as well as the threshold voltage, occurred at the lowest In molarity ratio, whereas the light sensitivity decreased with an increasing In molarity ratio. These observations correlate with the absorption results of the InO, ZnO, and IZO films shown in Figure 1b, indicating that ZnO in the IZO solution contributes to light sensitivity, whereas InO diminishes this effect. In addition, the leakage current, which depends on the photon energy, remained relatively invariant across different In molarity ratios. This suggests that the photoexcitation process was primarily attributed to the overall IZO semiconductor layers, not in the gate insulator.

The excitation mechanism in the IZO semiconductor due to optical energy is shown in Figure 1c, which is attributed to the EHP originating from the donor-like state density, N_VO_, distributed around the energy E_T_ level. In principle, holes from E_T_ do not significantly affect the electrical properties of amorphous semiconductors; however, excited electrons play a decisive role in TFT operation, such as the on-state current and threshold voltage. To interpret the density distribution of donor-like states corresponding to the threshold voltage shift, we employed the PECCS theory introduced by Lee et al. [33,34,35] and modified the relevant equations for a practical approach. The fundamental theory of PECCS can be explained using a simple charge–voltage (Q–V) relationship in the TFT operation. First, the excitation carrier density due to external energy is defined as

(1) $$n_{ph}\left(E\right)=\int_{E}^{E_{C}}N_{VO}\left(E\right)f_{D}\left(E\right)dE$$

where n_ph_(E) is the excited carrier density by the photon energy E = E_c_ − E_ph_ and N_VO_(E) is the density of the donor-like state near the valence band. The occupation Fermi–Dirac function f_D_(E) describes the probability of occupied donor-like states under energy level $E_{T}$. Using a straightforward simple charge-based metal-oxide-semiconductor (MOS) capacitor, the number of accumulated electrons near the oxide–semiconductor interface can be estimated as

(2) $$C_{ox}\left(V_{G}-V_{Th}\left(E\right)-V_{D}\right)=qn\left(E\right)d_{s}.$$

By incorporating the thickness of the IZO semiconductor d_s_ based on the charge-sheet approximation, the accumulated charges were equally distributed inside the semiconductor layer. C_ox_(V) and q are the area capacitance of the gate dielectric and electric charge of the electron, respectively. By differentiating Equation (2) with respect to the photon energy, the following can be obtained:

(3) $$\frac{dV_{Th}\left(E\right)}{dE}=-\frac{qd_{s}}{C_{ox}}\frac{dn\left(E\right)}{dE}.$$

For f_D_(E), the excited electron can only be generated when the electron is occupied at the donor-like states (i.e., f_D_(E) = 1 in Equation (1)) can be rearranged as follows:

(4) $$\frac{dV_{Th}\left(E\right)}{dE_{ph}}=-\frac{qd_{s}}{C_{ox}}N_{VO}\left(E_{ph}\right).$$

Subsequently, the donor-like state density N_VO_(E_ph_) is

(5) $$N_{VO}\left(E_{ph}\right)=-\frac{C_{ox}}{qd_{s}}\frac{dV_{Th}\left(E\right)}{dE_{ph}}.$$

In contrast to the derivation above, the fundamental definition of the threshold voltage operating in an accumulation without inversion is given by

(6) $$V_{Th}\left(E\right)=\phi_{ms}-\frac{Q_{eff}\left(E\right)}{C_{ox}}+\psi_{s,max}+\frac{Q_{G}}{C_{ox}}.$$

where Q_eff_(E) is the effective surface charge by electron accumulation at the semiconductor ψ_s.max_ is the surface potential due to the band bending and Q_G_ is the gate surface charge inside the gate dielectric. Considering that the leakage current of the IZO TFTs is not significantly affected by the photon energy, the gate charge Q_G_ can be reasonably negligible in terms of the photon energy. The threshold voltage differentiated with respect to the external energy E is

(7) $$\frac{dV_{Th}(E)}{dE}=-\frac{1}{C_{ox}}\frac{dQ_{eff}(E)}{dE}.$$

By introducing the surface density of the donor-like states D_VO_(E), the effective charge yields

(8) $$Q_{eff}\left(E\right)=q\int_{E}^{E_{C}}D_{VO}\left(E\right)f_{D}\left(E\right)dE$$

Given that f_D_(E) = 1 and E = E_C_ − E_ph_ in Equation (8) and substituting Q_eff_(E) into Equation (7), we obtain

(9) $$D_{VO}(E)=-\frac{C_{ox}}{q}\frac{dV_{Th}(E)}{dE}$$

In Equations (5) and (9), D_VO_(E) and N_VO_(E) represent the surface and bulk densities of the donor states, respectively. Under the assumption of the charge sheet approximation, where the charge is uniformly distributed within the thin film of the semiconductor channel, it can be expressed as D_VO_(E)/d_s_ = N_VO_(E), considering 20 nm of the thin IZO layer.

**Figure 3:**
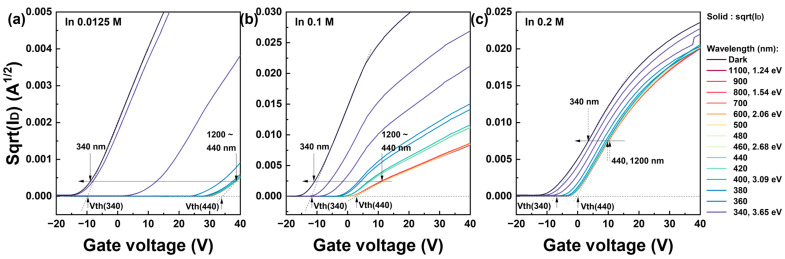
Square-root drain current vs. gate voltage graph of solution-processed IZO TFTs for threshold voltage extraction. The drain current was measured in the light wavelength range of 1200–340 nm and its drain voltage was 20 V, which is the saturation region. (**a**–**c**) represent the characteristics corresponding to different In molarity ratios; 0.0125 M, 0.1 M, and 0.2 M, all of which are based on a fixed Zn molarity ratio of 0.25 M.

To evaluate the electrical shift in the threshold voltage, a linear fitting of the square-root drain current was applied to extract the threshold voltage within the saturation voltage region (V_D_ = 20 V). Figure 3 presents the square-root drain current plotted against the gate voltage within a wavelength range of 1200–340 nm at 20 nm intervals. The threshold voltage was determined at the point where the linear fitting of the square-root current was plotted at the location where the maximum magnitude of the mobility intersects the *x*-axis. Additionally, Figure 3 depicts the photoelectrical characteristics corresponding to low, moderate, and high molarity ratios, respectively. The electrical parameters including the threshold voltage in terms of the In molarity ratio can also be found in our previous results [37]. The results based on the In molarity ratio are summarized in Supplementary Figure S3. In Figure 3, two distinct wavelength regions are observed: 1200–440 and 440–340 nm. First, in the 1200–440 nm range, there were minimal fluctuations in the current regardless of the In concentration. The most significant variation in the current occurred within the 440–340 nm range, leading to a negative shift in the threshold voltage. The largest threshold voltage shift was observed in Figure 3a for the lowest In concentration IZO TFT, indicating a predominant excitation by photon energy in this region. As the In molarity increased, the magnitude of the threshold voltage shift due to photon energy diminished, and this response was observed throughout the entire current region. As noted in the transfer curves, the fundamentals of the photoexcited carriers precisely follow the absorbance characteristics of the IZO film. By decreasing the In molarity ratio, the ZnO absorbance dominated the photosensitivity. Although not shown in these results, similar to the transfer curve, the photoexcited current recovered to its initial state as the wavelength decreased below 340 nm.

**Figure 4:**
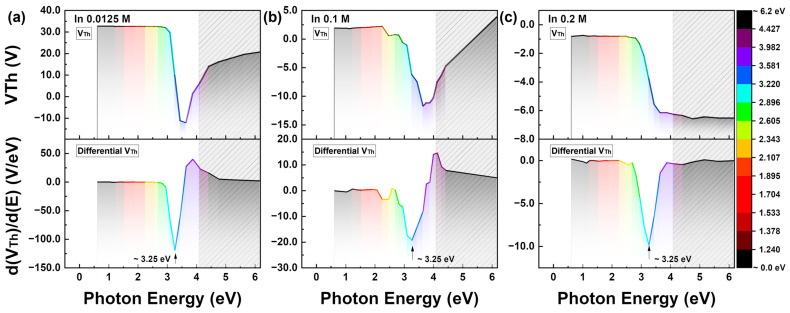
Threshold voltage as a function of photo energy and its differential result by the energy. The gray box area represents the out-of-range region where the output power of the light source decreased below 25% of its maximum magnitude. (**a**–**c**) depict the characteristics depending on the In molarity, which are 0.0125 M, 0.1 M, and 0.2 M.

Figure 4 illustrates the photon energy-dependent characteristics of V_Th_ and differentiated V_Th_ with respect to the In molar ratio. The results based on the In molarity ratio in Figure 4 are shown in Supplementary Figure S4. Photon energy in terms of the wavelength is derived using E_ph_ = hc/λ = 1239.8/λ, where h represents the Planck constant, c is the speed of light, and λ is the wavelength in nanometers (nm). All over the wavelength range of 1200–200 nm, the photon energy spans 1.03–6.19 eV. The gray box in the graph indicates the out-of-measurement region, where the source output power decreases by 25% from its maximum power, which is approximately half of its uniform power. The negative shifts in the threshold voltage should increase, because the valence band theoretically exists at energies greater than 3.65 eV as estimated from the absorbance of the IZO film. Although the output power started to decrease at 420 nm (~2.95 eV) and entered the out-of-range region beyond 280 nm (~4.43 eV), the measured peak characteristic at 3.26 eV is valid because the threshold voltage shift did not correspond to the decrease in the output source power. This can be explained by the fact that the maximum shift was attributed to the absorbance of ZnO at 380 nm. As discussed, the threshold voltage shift remains constant at 1200–440 nm (i.e., approximately 1.03–2.82 eV) and the significant changes are observed in the 440–280 nm range (i.e., approximately 2.82–4.42 eV). In the d(V_Th_)/d(E) results in Figure 4a–c, the peak change due to the energy was observed at 3.26 eV, regardless of the In molarity ratio, and the maximum magnitude was observed at the lowest In molarity ratio.

**Figure 5:**
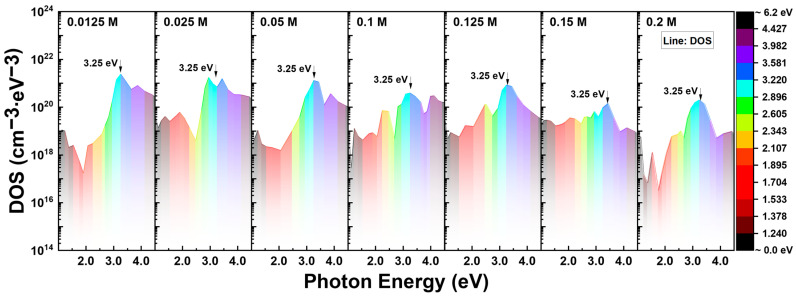
Density of donor-like state in the IZO semiconductor band gap as a function of the In molarity ratio, with a fixed Zn molarity ratio of 0.25 M. The DOS profiles were determined by the PECCS analysis method.

The surface DOS, denoted D_VO_, was quantitatively determined using Equation (9). The distributions of the donor-like states within the solution-processed IZO semiconductor are summarized in Figure 5. The discrete data distributions within the graph resulted from the transfer curves measured at 20 nm intervals. Additionally, it was difficult to precisely define the peak point of the E_T_ level; however, the maximum DOS was computed when irradiating IZO TFTs with 380 nm light (equivalent to ~3.26 eV). The on/off ratio of the DOS distribution, ranging from the lowest to the highest magnitude, remained at approximately 10^2^ for different In molarity ratios and no specific trend was identified. The relatively low on/off ratio of 10^2^ could be attributed to the linear-scale transition of the threshold voltage and minor variations in the 1200–440 nm region. Even with minimal V_Th_ fluctuations, log-scale variations could not be detected owing to threshold voltage extraction via linear fitting. Notably, the maximum density of donor-like states was observed at the lowest In concentration of 0.0125 M, and the maximum magnitude decreased monotonically as the Zn composition ratio decreased.

As confirmed by the results of the distribution of the donor-like state, the most significant advantage of the PESCCS analysis lies in its theoretical background and straightforward approach involving typical threshold voltage extraction. However, it is important to note that the measurement time significantly increases depending on the interval, which can lead to decreased accuracy owing to photocarrier accumulation. In detail, to measure the threshold voltage at 20 nm intervals within 100 nm range, even if it takes a minimum 10 s to measure each transfer curve, it takes more than 50 s to measure five data points. Considering the charge transport between trap states within the semiconductor, it is crucial to thoroughly discuss the generation or collapse of excited photocarriers because these processes significantly affect the resulting DOS distribution. Therefore, to calculate the DOS distribution precisely, it is necessary to consider a measurement method to reduce the time interval.

## 4. Spectroscopy-Induced Photo-Carrier Analysis

Photocurrent characteristics are subject to variations based on light irradiation. This can sometimes blur the distinction in photocarrier generation between the effects of photon energy and accumulation by prolonged irradiation. To quantitatively calculate the donor-like-state distribution in a solution-processed IZO semiconductor, it is crucial to restrict the impact of light wavelength by excluding the influence of light accumulation. The photocurrent of the solution-processed IZO semiconductor reflects the distribution of the occupied donor-like states at the E_T_ energy level, which lies below the E_F_ level. Theoretically, while the PECCS method takes a minimum of 10 s to obtain data for one point, the photocurrent measurement method takes only a few ms to measure data for one point. A quantitative calculation of the DOS using PIDS analysis is demonstrated by evaluating the photocurrent with respect to the light wavelength. The excited photocurrent I_D_ph_, which depends on the light irradiance, can be expressed as follows:

(10) $$I_{D\_ph}(E)=I_{D\_illumination}(E_{ph})-I_{D\_dark},$$

where I_D_illumination_(E_ph_) is the photoexcited drain current due to photon energy, and I_D_dark_ is the drain current measured in the dark state. The photocurrent, based on the approach of the electron drift current depending on the photon energy, is given by

(11) $$I_{D\_ph}\left(E\right)=q\mu_{FE}n_{ph}\left(E\right)\;\xi_{D}A.$$

Here, μ_FE_, n_ph_(E), and ξ_D_ denote the field-effect mobility, effective carrier density by photon energy, and the electric field between the source/drain electrodes, respectively. The cross-sectional area, A, is the product of the channel width (W) and semiconductor thickness (d_s_). The excited carrier density can then be rewritten as

(12) $$n_{ph}\left(E\right)=\frac{I_{D\_ph}(E)}{q\;\mu_{FE}\xi_{D}A}\;.$$

The photoexcited electrons through the EHPs are consistently generated at the R-G center, E_T_, and the electrons are elevated to the conduction band by the photon energy. The total free-carrier density, which is excited by the photon energy, can be evaluated by integrating the product of N_VO_ and f_D_(E) from E to E_C_:

(13) $$n_{ph}\left(E\right)=\int_{E}^{E_{C}}N_{VO}(E)f_{D}(E)dE.$$

By substituting Equation (12) into (13):

(14) $$\frac{I_{D\_ph}\left(E\right)}{q\;\mu_{FE}\xi A}=\int_{E}^{E_{C}}N_{VO}(E)f_{D}(E)dE.$$

By applying f_D_(E) = 1, the density of the donor-like states can be derived by differentiating both sides of the equation by energy as follows:

(15) $$\frac{1}{q\mu_{FE}\xi A}\left(\frac{dI_{D\_ph}(E)}{dE}\right)=N_{V_{O}}(E).$$

In Equation (15), the photocurrent I_D_ph_(E) is a function of photon energy. By differentiating the photoexcited current with respect to the energy, the density of the donor-like state below the E_F_ level can be quantitatively calculated. The PIDS analysis was devised to minimize the influence of time. The main advantage of the PIDS analysis is that it does not require a transfer curve to extract the threshold voltage. Therefore, the measured photocurrent spectrum can be directly applied to calculate the DOS distribution with minimal impact from the measurement interval.

**Figure 6:**
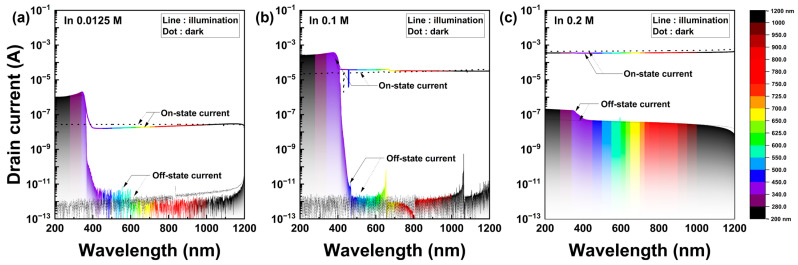
Photo-excited current spectroscopy of the solution-processed IZO TFTs. The on- and off-state current are characteristics measured at V_G_ = −5 V and 20 V, respectively, with an applied drain voltage V_D_ = 20 V. The measurement speed and interval of the light wavelength were 1 nm/s and 5 nm, respectively. (**a**–**c**) indicate the photocurrent spectroscopy corresponding to the In molarity ratios of 0.0125 M, 0.1 M, and 0.2 M.

Figure 6 shows the drain current characteristics of the solution-processed IZO TFTs as a function of the light wavelength at a speed of 2 nm/s depending on the wavelength. The operating gate voltages for the on- and off-state currents were determined from the transfer curves, whereas the drain voltage was derived from the output curve under saturation conditions. Additionally, Figure 6 depicts the photocurrent spectra in accordance with the In molarity ratio:0.0125 M (low), 0.1 M (moderate), and 0.2 M (high). More specific photocurrent spectra based on the In molarity ratio are shown in Supplementary Figure S5. As shown in Figure 6, the photocurrent of the solution-processed IZO TFTs exhibited a drastic increase starting at 420 nm, regardless of the In molarity ratio. This photocurrent excitation mechanism was directly correlated with the absorbance of the IZO film. Notably, the photoexcitation mechanism appeared to be independent of the gate bias, implying that the impact of the surface band bending on the TFT was not critical to the R-G process, even across a large portion of the semiconductor channel formed in the film. Figure 6a,b shows that the generated photocarriers dominate the drain current over the on-state current. However, this effect diminished as the In molarity ratio increased (Figure 6c). In particular, the photogeneration effect was only detected in the off state for high In molarity ratios. The magnitude of the photocurrent is maximum at a moderate In molarity ratio of 0.1 M and minimum at 0.2 M, the highest In molarity ratio.

**Figure 7:**
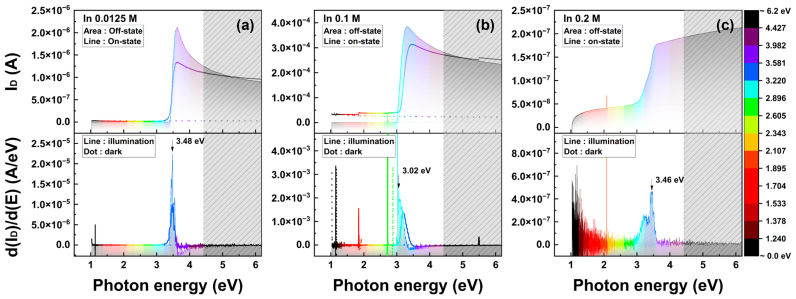
Characteristics of the photo-excited current and its differential response to energy, depending on the photon energy. (**a**–**c**) graphs are measured at 0.0125 M, 0.1 M, and 0.2 M of the In molarity ratio. The gray box inside the graphs represents the out-of-range power region.

Figure 7 shows the photocurrent and differential drain current as functions of the photon energy. The photon energy, depending on the wavelength, is converted using E = hν. The In molarity dependent characteristics according to 0.0125 M, 0.1 M, and 0.2 M are analyzed in Figure 7a–c. In Figure 7c, the drain current and d(I_D_)/d(E) results in the off-state region are highlighted by neglecting the results in the on-state region. Photoexcitation was not observed in the on-state characteristics of the TFT with high In molarity ratios. According to the various In molarity ratios, extensive graphs of the data in Figure 7 are shown in Supplementary Figure S6. In Figure 7, the maximum d(I_D_)/d(E) peaks obtained with the range of 3.0 to 3.5 eV and the photon energy where the peak observed is slightly lower in 0.1 M of In molarity. The energy level of the R-G center, E_T_, can be approximated using the peak point of the d(I_D_)/d(E) graph. A clearer peak is observed in Figure 7a,b and the magnitude of the differential I_D_ is marked at the 0.1 M of the In molarity ratio.

**Figure 8:**
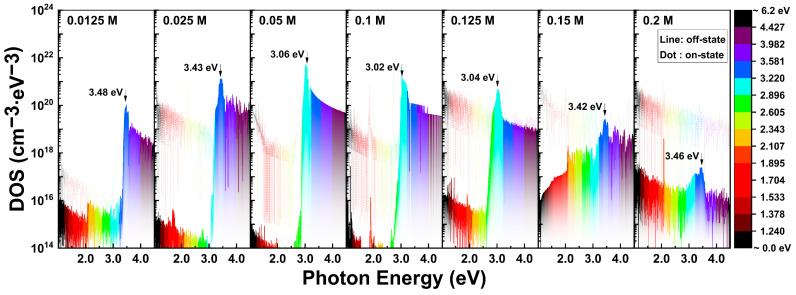
Calculated DOS through PIDS analysis methods with respect to the In molarity ratios.

In the PIDS calculations using Equation (15), the distribution of the donor-like state density depending on the In molar ratio is summarized in Figure 8. To calculate Equation (15), the maximum field-effect mobility, μ_FE_, obtained from the transfer curve is applied to the TFT characteristics. The maximum field-effect mobilities used in this analysis are 0.033, 0.092, 0.89, 0.77, 0.46, 5.22, and 2.73 cm^2^V^−1^s^−1^. This can be applicable to off-state currents because the photo-excited current will eventually operate in an on-state region. As shown in Figure 8, the distribution of the donor-like states was clearly estimated through photocurrent analysis. Especially, traps existing in 3.0–3.5 eV can be predominantly detected using PIDS analysis. The magnitude of the DOS in terms of the In molarity is highest at 0.1 M and decreases as the In molarity increases or decreases. The lowest magnitude of DOS is observed in the highest In molarity of 0.2 M. Although no particular tendency in the peak position was analyzed, the energy positions of the R-G center, E_T_, were distributed between 3.0 eV and 3.5 eV, and were measured to be relatively low in the moderate-doped TFTs of 0.05 M, 0.1 M, and 0.125 M. We speculated on these results that as the reactivity to the light increased, the peak position appeared more rapidly.

The main advantages of the PIDS measurement and analysis method are its ability to provide intuitive data and enable the rapid assessment of donor-like states below the E_F_ energy level. As discussed previously, the accuracy and measurement speed can be effectively improved by employing photoexcited current analysis. Including the PECCS analysis, the distributions of the DOS near the edge of the E_C_ and E_V_ levels cannot be measured using photon energy analysis, as unoccupied states over the E_F_ energy cannot be measured using photon energy, and the optical power of the light source begins to diminish beyond 3.5 eV. However, this limitation in optical analysis can be overcome by employing thermal analysis and ensuring a uniform output power in the out-of-range regions.

**Figure 9:**
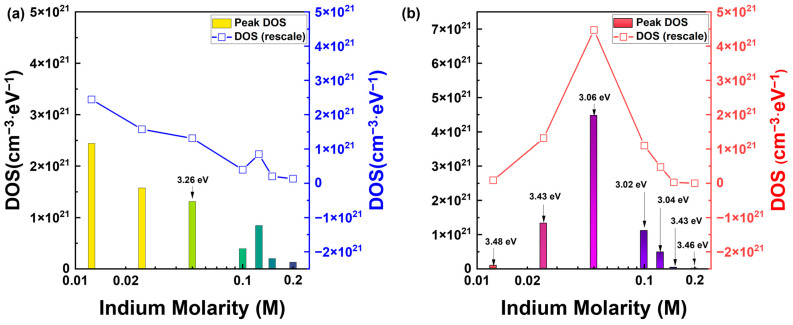
Calculated maximum magnitude of DOS distribution with respect to In molarity ratio. (**a**,**b**) present the highest density of the donor-like state derived by the PECCS and PIDS analyses, respectively. The bars indicate the magnitude of the peak DOS, whereas the line describes the trends depending on the In molarity ratio. The right side of the *y*-axis is rescaled to highlight the tendency.

The interpretations of the results presented in Figure 5 and Figure 8 through the PECCS and PIDS analyses are controversial. Figure 9 indicates that the maximum DOS is a function of the In molarity ratio. Figure 9a shows the results obtained through PECCS calculations, whereas Figure 9b shows the results obtained through PIDS calculations. It should be noted that the calculated DOS resulting from the PECCS decreases with increasing In molarity, whereas the PIDS calculation is the highest at 0.1 M. Although differences in the calculations can occur depending on the applied parameters, the differences in trends require a clear interpretation to understand the mechanisms. It is difficult to find evidence of the differences in the trends based on the threshold voltage or current analysis methods. Eventually, the cause of the photoexcited carriers can be attributed to the ZnO composition ratio within the IZO solution, with higher ZnO ratios leading to a greater photoresponse. This can be understood through a similar study where metal-oxide nanoparticles, such as TiO_2_ consistently generate EHPs at the surface of the particles under light irradiation, due to the their imperfect atomic structures [38,39,40,41]. Therefore, although the results in Figure 9a are reasonably predictive, those in Figure 9b may lack validity.

**Figure 10:**
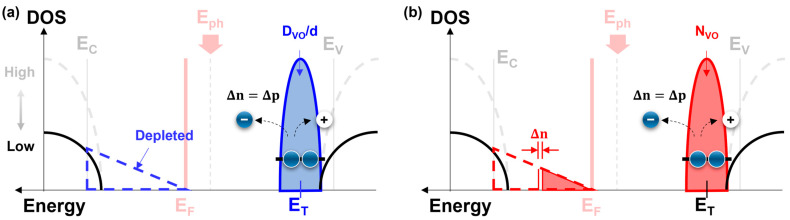
Schematic illustration of the calculated donor-like state distribution derived from (**a**) PECCS and (**b**) PIDS analysis.

For a low photocurrent at a low In molarity ratio, we demonstrated various aspects of the origin of the reduction in the TFTs. Despite the high ZnO composition ratio, our interpretation of the low photocurrent is as follows. Figure 10 shows the DOS distribution of the IZO semiconductor in a band diagram. Figure 10a shows the DOS analysis using the PECCS method, and Figure 10b outlines the PIDS method. In this diagram, the gray band represents the DOS distribution with a high density of acceptor-like states, whereas the black line represents the band diagram with a low density of acceptor-like states. The EHPs generated through photoexcitation are transported toward the E_C_ and expressed as the photocurrent of the TFT. Electrons at the E_T_ level are excited by the photon energy and gradually fill the unoccupied states above the E_F_ level. However, if the DOS states at E_C_ are lower than the N_VO_ states at E_T_, the density of the acceptor-like states to be filled is limited, preventing the accommodation or occupation of excited carriers. Supporting information on the distribution of the acceptor-like states according to the In molarity ratio can be found in our previous study [42]. The influence of the low density of the acceptor-like states is primarily expressed at the PIDS analysis, because the PIDS immediately reflects the response in excitation to Δn, as shown in Figure 10b. However, in the analysis shown in Figure 10a, the transfer curves should be scanned with respect to the wavelength intervals for threshold voltage extraction. Under the same conditions, the acceptor-like states distributed in Figure 10a were continuously depleted by the gate-voltage sweep, allowing substantially more space for the excited electrons to fill. Consequently, in Figure 10a, the triangular region extends the photocurrent variation, whereas in Figure 10b, the region corresponds to n. It is speculated that the difference in the trends between the two analysis methods can be attributed to the measurement approaches.

The misleading PIDS analysis in the case of a low In molarity in IZO semiconductors can be attributed to their inability to accommodate a high density of N_VO_ at the E_C_ level. Herein, the DOS near E_C_ is closely correlated to the conductivity of the drain current based on the conductivity σ = qμn. In Equation (11), the decrease in current due to low conductivity can be carried out by carriers but may also be caused by mobility. The influence of the In mobility on μ_FE_ can be observed from the transfer characteristics of the solution-processed IZO TFTs according to the In molarity ratio. In Equation (15), the maximum field-effect mobility was used, ranging from 0.033 to 5.22 cm^2^V^−1^s^−1^, for N_VO_ calculations based on the In molarity ratio. The field-effect mobility, μ_FE,_ and maximum μ_FE_max_ used in this analysis is:

(16) $$\mu_{FE}\left(V_{G}\right)=\frac{2}{C_{ox}W/L}{\left(\frac{\partial \sqrt{I_{Dsat}}}{\partial V_{G}}\right)}^{2},\;\;\mu_{FE\_max}=\frac{2}{C_{ox}W/L}{\left.{\left(\frac{\partial \sqrt{I_{Dsat}}}{\partial V_{G}}\right)}^{2}\right|}_{g_{m}=g_{m\_max}}.$$

**Figure 11:**
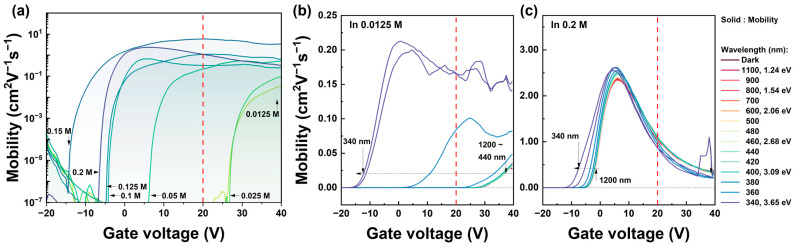
Gate voltage dependent characteristics of field-effect mobility graph. (**a**) Log-scale field-effect mobility as a function molarity ratio in dark state. (**b**,**c**) Gate-voltage-dependent field-effect mobility characteristics under illumination conditions, measured at 0.0125 M and 0.2 M of In molarity ratio.

The I_Dsat_ is the drain current of TFT at the saturation condition, and g_m_ and g_m_max_ are the transconductance and maximum transconductance of the square-root drain current, respectively, where $g_{m}=\partial \sqrt{I_{Dsat}}$/$\partial V_{G}$. Figure 11 shows the field-effect mobility characteristics as functions of the gate voltage. Figure 11a illustrates μ_FE_ with respect to the In molarity ratio under dark-state conditions, whereas Figure 11b,c show μ_FE_ under the illumination states, measured at 0.0125 M and 0.2 M of In molarity, respectively. The detailed μ_FE_ according to the molarity ratio are summarized in Supplementary Figure S7. For the PIDS measurements in this investigation, V_G_ = 20 V and V_D_ = 20 V were evaluated for application in the on-state condition, as indicated by the red dashed lines in the graphs. The maximum μ_FE_ remains effective over the on-state region, so it can be acceptable to apply. Nevertherless, to standardize the gate-field effect on TFTs as a function of In concentration, it is necessary to fix the gate voltage as V_G_ = 20 V. For cases with In molarity greater than 0.05 M (0.05 M, 0.1 M, 0.125 M, 0.15 M, and 0.2 M), the maximum μ_FE_ with respect to the gate voltage are almost comparable to V_G_ = 20 V. However, for low In molarity ratios, even at the V_G_ = 20 V bias, the μ_FE_ is significantly lower than 10^6^ compared to the maximum magnitude. Based on the fact that the I_D_ current of TFT with 0.2 M is significantly lower than 10^4^ compared to the I_D_ current with 0.0125 M, it is difficult to apply the same condition of μ_FE_ to all devices for interpretation, especially for low In mobility of 0.0125 M and 0.025 M. Based on this speculation, in the modified PIDS calculations, the maximum μ_FE_ or the mobility at V_G_ = 20 V are modified instead of those mobility for the calculation of Equation (15). In particular, compared with Figure 8, for low In molarities of 0.0125 M and 0.025 M, a correction factor was applied considering the characteristics of the on-state mobility differ by more than 10^6^ from those of the off-state mobility. This correction factor was approximated from the μ_FE_ magnitude under photo-excited conditions. The maximum field-effect mobility (μ_FE_ at V_G_ = 20 V) and the modified μ_FE_ results are summarized in Table 2. Although there is a difference in magnitude, in both the mobility results of Figure 11a and Table 2, it showed that the threshold voltage shifts in a negative direction and the mobility increases as the In molarity increases.

**Figure 12:**
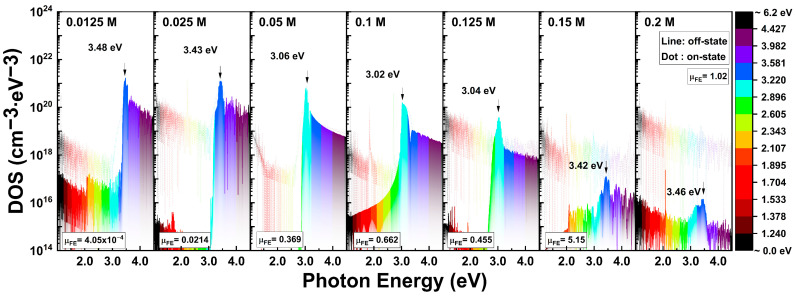
Modified distributions of density of donor-like state through the PIDS analysis with respect to the In molarity ratio. Field-effect mobility μ_FE_ indicates the modified magnitude for the DOS calculation.

The recalculated donor-like-state distributions based on the modified mobilities listed in Table 2 are shown in Figure 12. Figure 12 depicts the distribution of the donor-like states as a function of the photon energy. Consequently, similar to the results obtained through the PECCS analysis, the DOS distribution gradually decreased with an increasing In concentration, which is considered a more reasonable explanation. In previous researches, the DOS near the valence band has been reported as oxygen vacancies, which resulting from weak bonds and defects in the metal–oxide atomic bonding structures [43,44,45]. Additionally, it has been noted that as the Zn-O atomic ratio increases, the amorphous features become stronger, and a higher ratio of In-O atomic bonds lead to an increase in conductivity. In other words, as the ratio of Zn-O atomic bonds increased, the amorphous random network of the semiconductor increased, while the conductivity decreased. In this study, an enhancement in the photo-reactivity of solution-processed IZO TFTs was observed with higher molarity ratio of Zn-O bonds. Through this demonstration, the origin responsible for generating carriers under the photon energy is believed to be the ZnO particles, and the defects in the Zn-O atomic bonding structure act as R-G centers, similar to the metal-oxide photoreactive materials. This photosensitivity decreased as the In composition increased, and the R-G center energy, E_T_, distributed in the range of 3.0 to 3.5 eV without significant trends. The photocurrent behavior maximizes the variations in the off-state current, generating photocarriers that surpass the amount of the on-state current depending on the Zn:In ratio.

As discussed, the advantages of the PECCS analysis lie in its theoretical background and validity through electrical parameter analysis, whereas the strengths of the PIDS method include precise and intuitive data evaluation and rapid measurement speed. In particular, PIDS analysis is effective for log-scale DOS analysis based on photocurrents and can drastically reduce measurement intervals, as it does not require transfer curves for extraction. In this study, we proposed an effective PIDS methodology for detecting the DOS distribution near the valence band edge using photon energy. However, as mentioned in this paper, its application must consider the fundamental properties of the material. Therefore, by leveraging the complementary strengths of both the PECCS and PIDS methods to estimate the distribution of the band gap states, these analyses can become more effective tools for understanding semiconductor fundamentals in the near future.

## 5. Conclusions

In conclusion, this study provided a comprehensive evaluation of the density of donor-like state distributions in solution-processed IZO TFTs using photon energy irradiation. The primary focus was on quantitatively assessing the distribution of these donor-like states in IZO semiconductors, with particular attention to their calculation methods using electrical properties, such as the threshold voltage and photocurrent. Two calculation methods, PECCS and PIDS, were employed to estimate the density of the donor-like states. The results of the DOS distribution through photocurrent calculations indicate that as the In molarity ratio increases, the photo-reactivity decreases, leading to a reduction in the DOS magnitude. The decreased DOS distributions depending on the In molarity ratios suggest that the bonding structure, characterized by weak bonds and oxygen vacancies within the Zn-O bonds, acts as the primary source of the R-G centers. The results obtained in this study deepen our understanding of the charge transport mechanisms in IZO semiconductors. Furthermore, the calculation methods are not only academically valuable but also have practical implications. They offer the potential for improved performance and reliability of solution-processed IZO TFTs. The insights gained here hold promise for addressing stability concerns, such as NBIS. By investigating the fundamental properties of IZO semiconductors and their donor-like states, this study contributes significantly to the advancement of semiconductor materials.

## Figures and Tables

**Table 1:** Molarity and atomic mass ratio of IZO solution.

Molecular Weight (g/mol)	Molarity of Solution (M)						
300.83 (Indium nitrate hydrate)	0.0125	0.025	0.05	0.1	0.125	0.15	0.2
189.40 (Zinc nitrate hydrate)	0.25						
Atomic weight ratio of Zn:In	1:0.086	1:0178	1:0.350	1:0.706	1:0.883	1:1.055	1:1.368

**Table 2:** Maximum magnitude of field-effect mobility, the field-effect mobility under the fixed bias condition, and modified field-effect mobility by photon energy in terms of the In molarity ratio.

In Molarity Ratio (M)		0.0125	0.025	0.05	0.1	0.125	0.15	0.2
Zn Molarity Ratio (M)		0.25						
**Mobility** **(cm^2^V^−1^s^−1^)**	**Maximum field-effect mobility**	0.033	0.092	0.89	0.77	0.46	5.22	2.23
	**Field-effect mobility at V_G_ = 20 V and V_D_ = 20 V**	1.72 × 10^−9^	2.0 × 10^−10^	0.37	0.66	0.46	5.15	1.02
	**Modified field-effect mobility**	* 4.05 × 10^−4^	* 0.021	0.37	0.66	0.46	5.15	1.02

* These values have been modified from the data in the previous row.

## Data Availability

The research data presented in this study are available on request from the corresponding author.

## Supplementary Materials

The following supporting information can be downloaded at: https://www.mdpi.com/article/10.3390/nano13232986/s1, Figure S1. UV/Vis. light absorption characteristics of solution-processed metal-oxide films fabricated with different Zn:In molarity ratios; Figure S2. Transfer characteristics of solution-processed IZO TFTs under a light wavelength irradiation. The drain voltage is biased at 20 V, representing the saturation condition. (a–g) depict the transfer curves for seven different In molarity ratios: (a) 0.0125 M, (b) 0.025 M, (c) 0.05 M, (d) 0.1 M, (e) 0.125 M, (f) 0.15 M, and (g) 0.2 M, while maintaining a constant Zn molarity of 0.25 M; Figure S3. Threshold voltage extraction in solution-processed IZO TFTs through square-root drain current versus gate voltage analysis. Drain current was measured under illumination with light wavelength ranging from 1200 to 340 nm. (a–g) correspond to the distinct In molarity ratios: (a) 0.0125 M, (b) 0.025 M, (c) 0.05 M, (d) 0.1 M, (e) 0.125 M, (f) 0.15 M, and (g) 0.2 M, with a consistent Zn molarity ratio of 0.25 M; Figure S4. Threshold voltage characteristics in response to varying photon energies and their respective differentials. (a–g) illustrate these characteristics for different In molarity ratios, corresponding to 0.0125 M, 0.025 M, 0.05 M, 0.1 M, 0.125 M, 0.15 M, and 0.2 M, respectively; Figure S5. Photocurrent spectroscopy analysis of solution-processed IZO TFTs with respect to the different In molarity ratios. (a–g) represent the photocurrent spectroscopy results for In molarity ratios of 0.0125 M, 0.025 M, 0.05 M, 0.1 M, 0.125 M, 0.15 M, and 0.2 M, respectively; Figure S6. Differential characteristics of photocurrent depending on photon energy and In molarity ratios. The graphs (a–g) correspond to different In molarity ratios of 0.0125 M, 0.025 M, 0.05 M, 0.1 M, 0.125 M, 0.15 M, and 0.2 M, respectively; Figure S7. Gate-voltage dependent field-effect mobility as a function of In molarity ratio under light wavelength irradiation. (a–g) Field-effect mobilities were calculated from the square root of drain current of IZO TFTs at different In molarity ratios: 0.0125 M, 0.025 M, 0.05 M, 0.1 M, 0.125 M, 0.15 M, and 0.2 M.
