# Supplementary material for: Investigation of Donor-like State Distributions in Solution-Processed IZO Thin-Film Transistor through Photocurrent Analysis
Source: Nanomaterials (Basel). 2023 Nov 21;13(23):2986. doi: 10.3390/nano13232986 (PMC10707932; doi:10.3390/nano13232986)
Supplement: Supplementary file 1 [file nanomaterials-13-02986-s001.zip › nanomaterials-2685994-supplementary.pdf]

Figure S1. UV/Vis. light absorption characteristics of solution-processed metal-oxide films fabricated with different Zn:In molarity ratios.

Figure S2. Transfer characteristics of solution-processed IZO TFTs under a light wavelength irradiation. The drain voltage is biased at 20 V, representing the saturation condition. (a)–(g) depict the transfer curves for seven different In molarity ratios: (a) 0.0125 M, (b) 0.025 M, (c) 0.05 M, (d) 0.1 M, (e) 0.125 M, (f) 0.15 M, and (g) 0.2 M, while maintaining a constant Zn molarity of 0.25 M.

Figure S3. Threshold voltage extraction in solution-processed IZO TFTs through square-root drain current versus gate voltage analysis. Drain current was measured under illumination with light wavelength ranging from 1200 to 340 nm. (a)–(g) correspond to the distinct In molarity ratios: (a) 0.0125 M, (b) 0.025 M, (c) 0.05 M, (d) 0.1 M, (e) 0.125 M, (f) 0.15 M, and (g) 0.2 M, with a consistent Zn molarity ratio of 0.25 M.

Figure S4. Threshold voltage characteristics in response to varying photon energies and their respective differentials. (a)–(g) illustrate these characteristics for different In molarity ratios, corresponding to 0.0125 M, 0.025 M, 0.05 M, 0.1 M, 0.125 M, 0.15 M, and 0.2 M, respectively.

Figure S5. Photocurrent spectroscopy analysis of solution-processed IZO TFTs with respect to the different In molarity ratios. (a)–(g) represent the photocurrent spectroscopy results for In molarity ratios of 0.0125 M, 0.025 M, 0.05 M, 0.1 M, 0.125 M, 0.15 M, and 0.2 M, respectively.

Figure S6. Differential characteristics of Photocurrent depending on photon energy and In molarity ratios. The graphs (a)–(g) correspond to different In molarity ratios of 0.0125 M, 0.025 M, 0.05 M, 0.1 M, 0.125 M, 0.15 M, and 0.2 M, respectively.

Figure S7. Gate-voltage dependent field-effect mobility as a function of In molarity ratio under light wavelength irradiation. (a)–(g) Field-effect mobilities were calculated from the square-root of drain current of IZO TFTs at different In molarity ratios: 0.0125 M, 0.025 M, 0.05 M, 0.1 M, 0.125 M, 0.15 M, and 0.2 M.

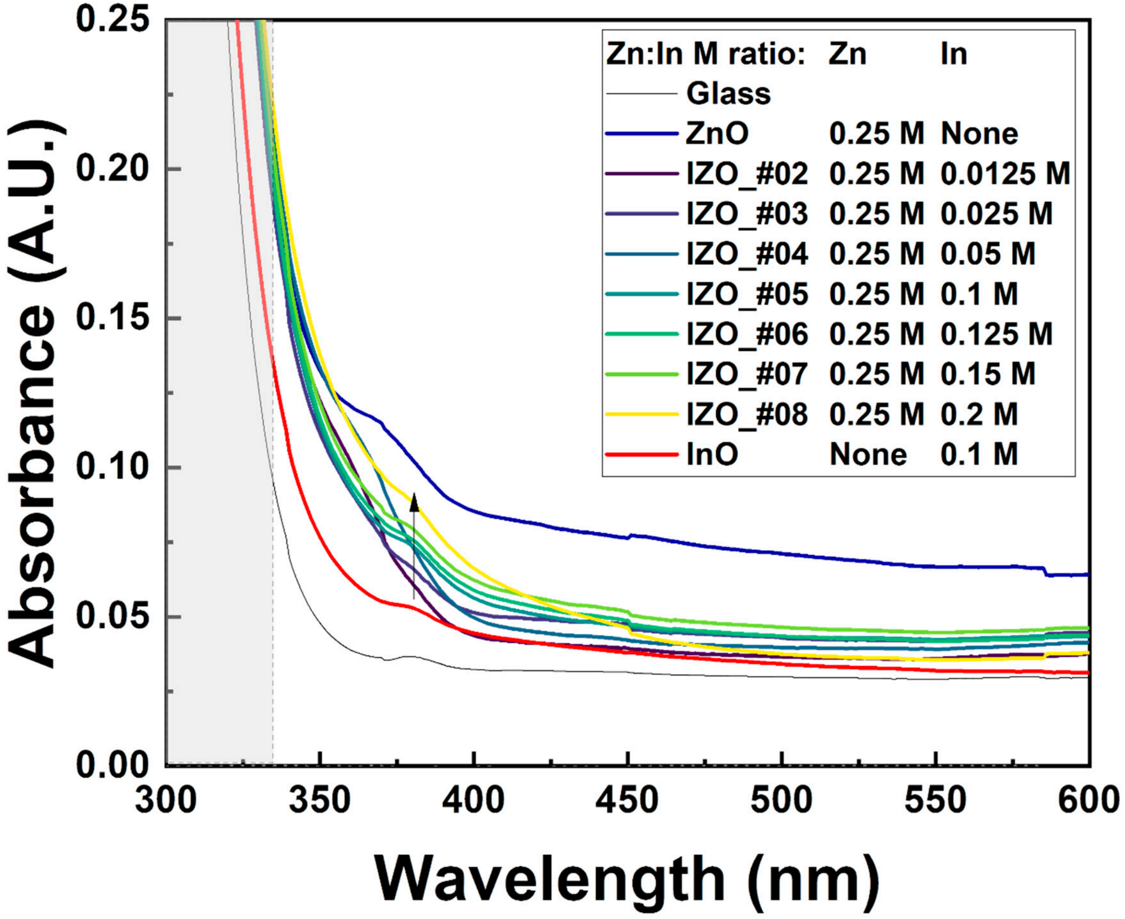

Zn:In M ratio: Zn In
Glass
ZnO 0.25 M None
IZO_#02 0.25 M 0.0125 M
IZO_#03 0.25 M 0.025 M
IZO_#04 0.25 M 0.05 M
IZO_#05 0.25 M 0.1 M
IZO_#06 0.25 M 0.125 M
IZO_#07 0.25 M 0.15 M
IZO_#08 0.25 M 0.2 M
InO None 0.1 M
Absorbance (A.U.)
0.25
0.20
0.15
0.10
0.05
0.00
300
350
400
450
500
550
600
Wavelength (nm)

(Figure S1)

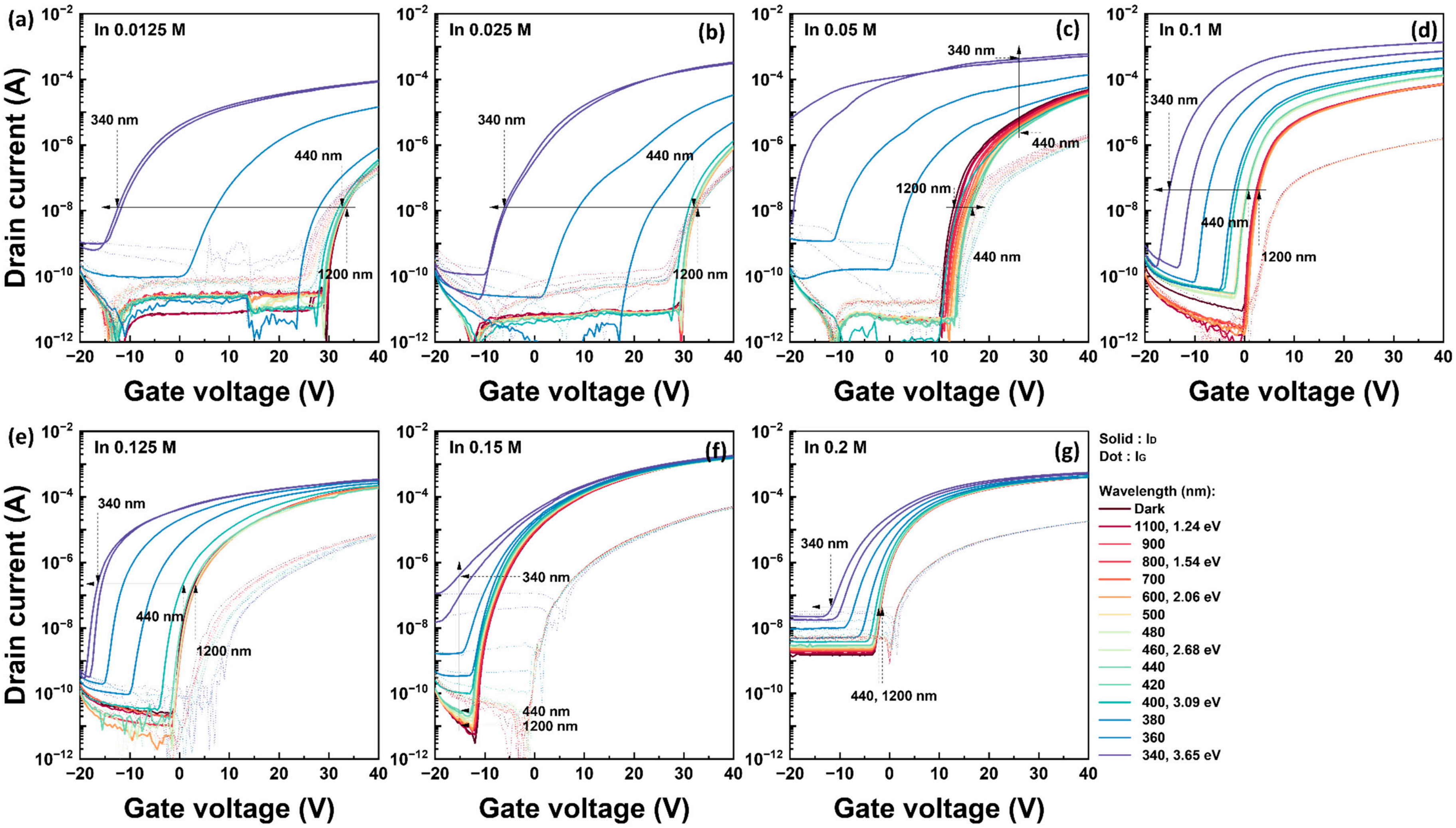

(a)
In 0.0125 M
340 nm
440 nm
1200 nm
(b)
In 0.025 M
340 nm
440 nm
1200 nm
(c)
In 0.05 M
340 nm
440 nm
1200 nm
440 nm
(d)
In 0.1 M
340 nm
440 nm
1200 nm
(e)
In 0.125 M
340 nm
440 nm
1200 nm
(f)
In 0.15 M
340 nm
440 nm
1200 nm
(g)
In 0.2 M
340 nm
440, 1200 nm
Drain current (A)
Gate voltage (V)
Solid : ID
Dot : IG
Wavelength (nm):
Dark
1100, 1.24 eV
900
800, 1.54 eV
700
600, 2.06 eV
500
480
460, 2.68 eV
440
420
400, 3.09 eV
380
360
340, 3.65 eV

(Figure S2)

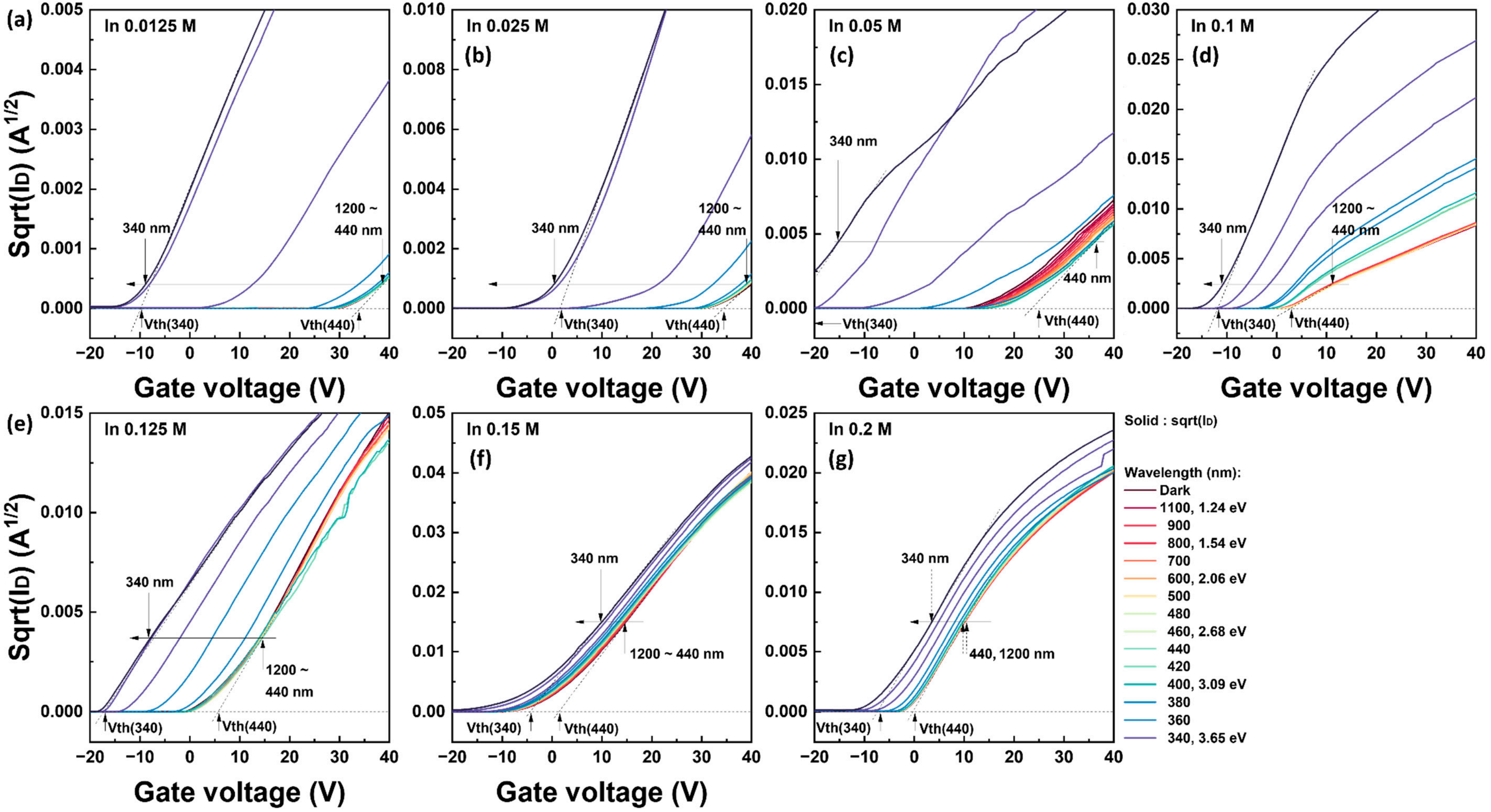
(a) In 0.0125 M
(b) In 0.025 M
(c) In 0.05 M
(d) In 0.1 M
(e) In 0.125 M
(f) In 0.15 M
(g) In 0.2 M
Sqrt(ID) (A1/2)
Gate voltage (V)
340 nm
1200 ~ 440 nm
440 nm
440, 1200 nm
Vth(340)
Vth(440)
Solid : sqrt(ID)
Wavelength (nm):
Dark
1100, 1.24 eV
900
800, 1.54 eV
700
600, 2.06 eV
500
480
460, 2.68 eV
440
420
400, 3.09 eV
380
360
340, 3.65 eV

(Figure S3)

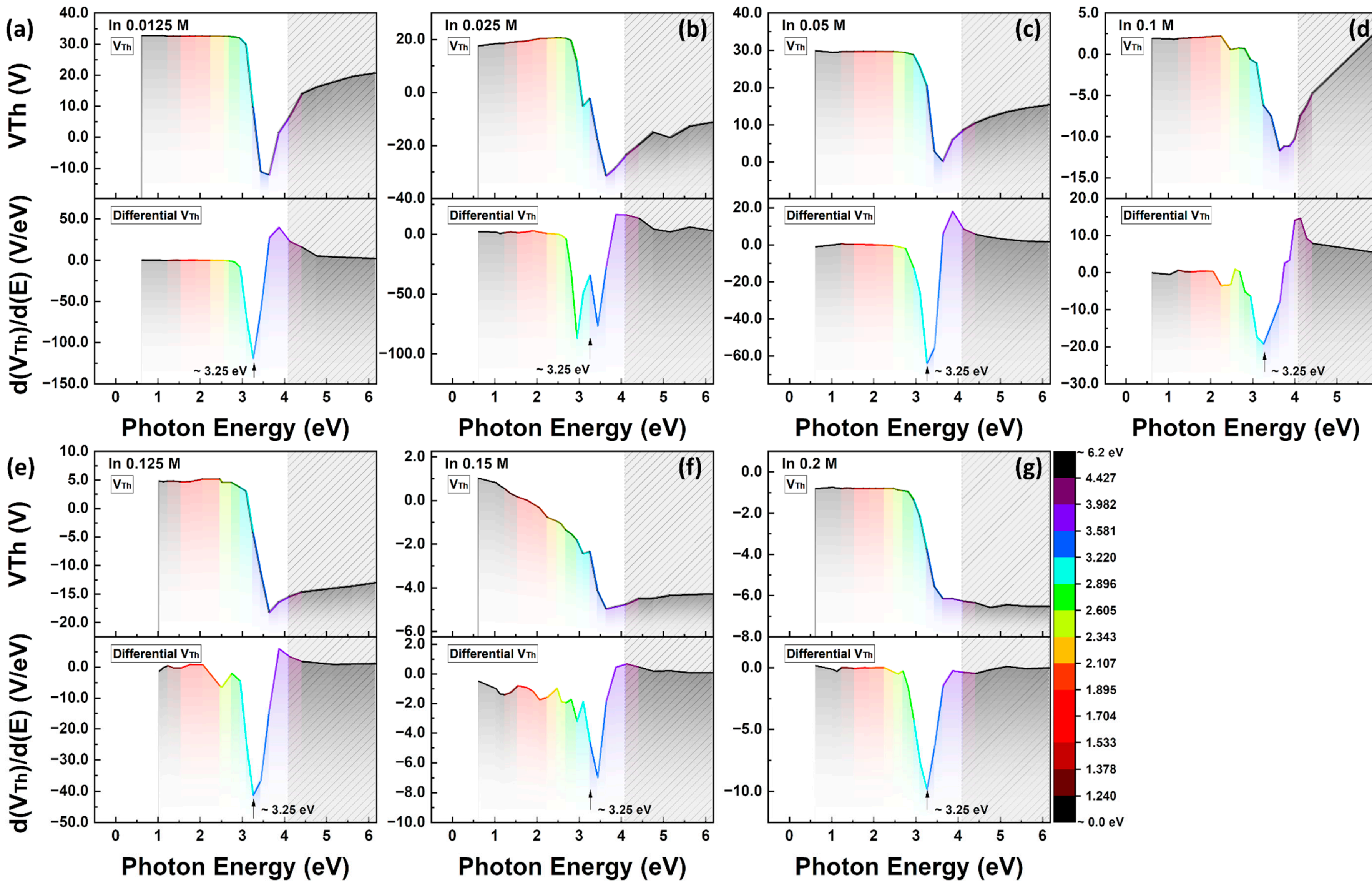

(a)
In 0.0125 M
VTh
Differential VTh
~ 3.25 eV
(b)
In 0.025 M
(c)
In 0.05 M
(d)
In 0.1 M
(e)
In 0.125 M
(f)
In 0.15 M
(g)
In 0.2 M
VTh (V)
d(VTh)/d(E) (V/eV)
Photon Energy (eV)
~ 6.2 eV
4.427
3.982
3.581
3.220
2.896
2.605
2.343
2.107
1.895
1.704
1.533
1.378
1.240
~ 0.0 eV

(Figure S4)

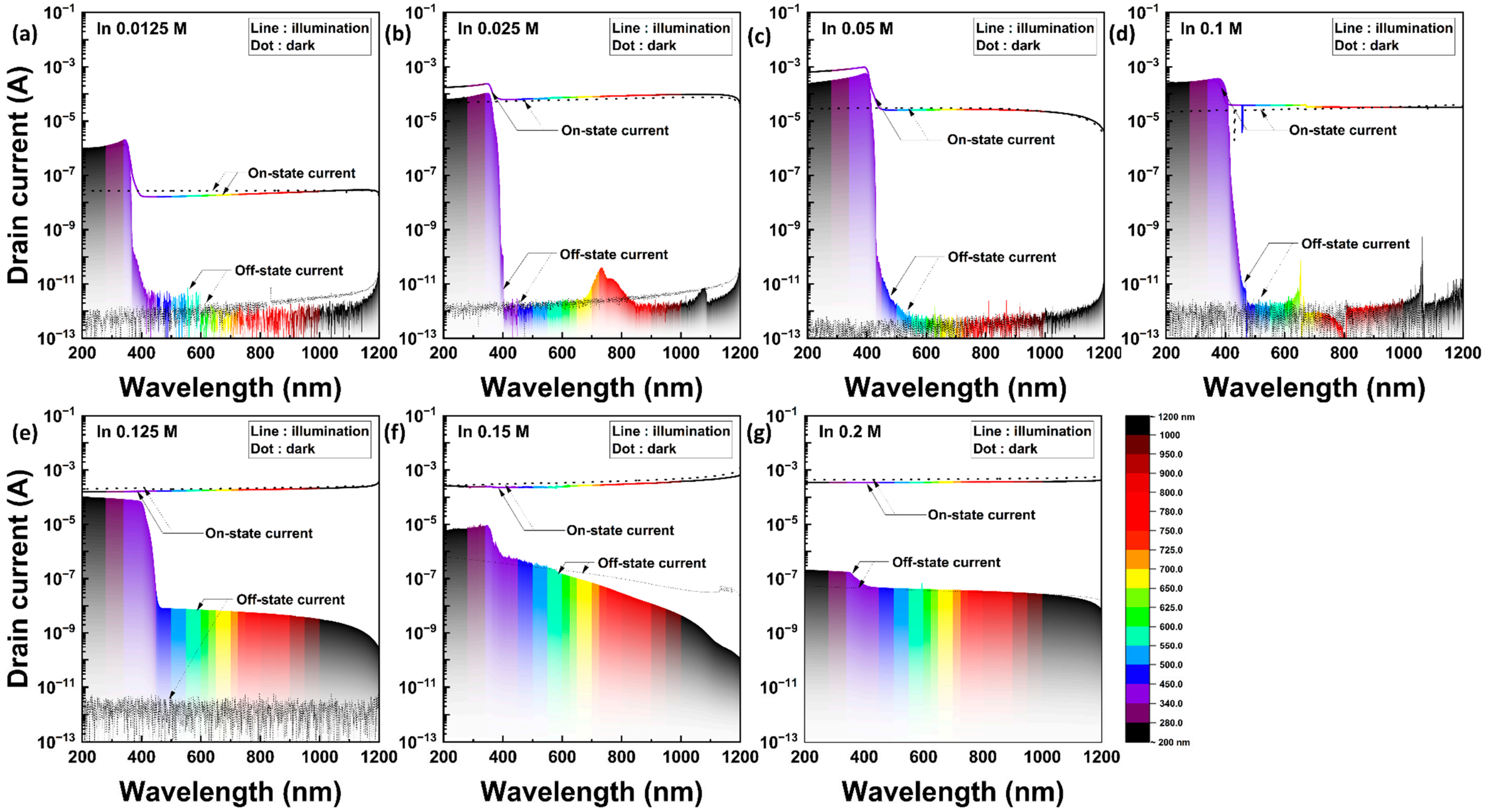
(a)
In 0.0125 M
Line : illumination
Dot : dark
On-state current
Off-state current
(b)
In 0.025 M
On-state current
Off-state current
(c)
In 0.05 M
On-state current
Off-state current
(d)
In 0.1 M
On-state current
Off-state current
(e)
In 0.125 M
On-state current
Off-state current
(f)
In 0.15 M
On-state current
Off-state current
(g)
In 0.2 M
On-state current
Off-state current
Drain current (A)
Wavelength (nm)
1200 nm
1000
950.0
900.0
800.0
780.0
750.0
725.0
700.0
650.0
625.0
600.0
550.0
500.0
450.0
340.0
280.0
200 nm

(Figure S5)

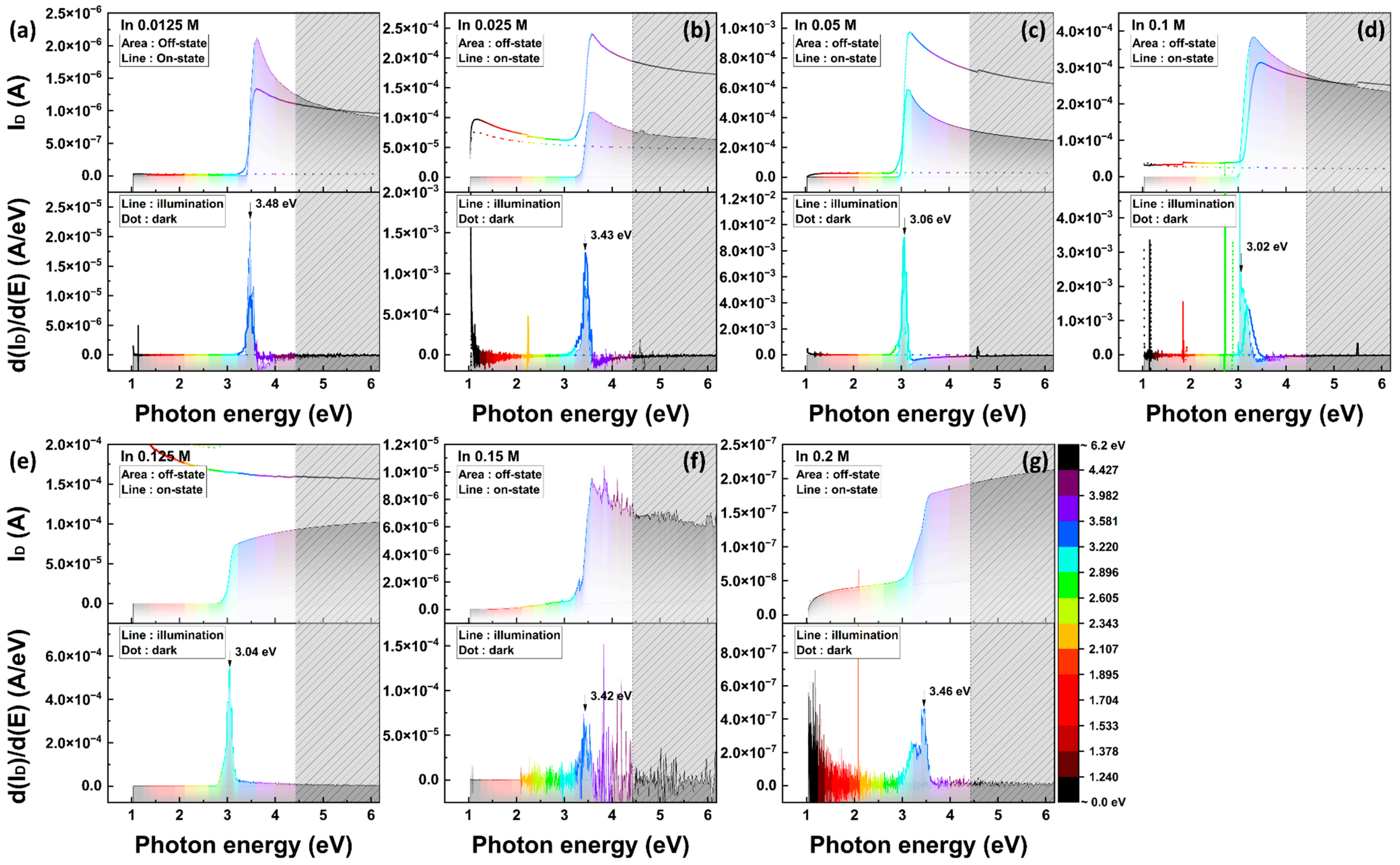

(a)
In 0.0125 M
Area : Off-state
Line : On-state
3.48 eV
(b)
In 0.025 M
Area : off-state
Line : on-state
3.43 eV
(c)
In 0.05 M
3.06 eV
(d)
In 0.1 M
3.02 eV
(e)
In 0.125 M
3.04 eV
(f)
In 0.15 M
3.42 eV
(g)
In 0.2 M
3.46 eV
Line : illumination
Dot : dark
I_D (A)
d(I_D)/d(E) (A/eV)
Photon energy (eV)
~ 6.2 eV
4.427
3.982
3.581
3.220
2.896
2.605
2.343
2.107
1.895
1.704
1.533
1.378
1.240
~ 0.0 eV

(Figure S6)

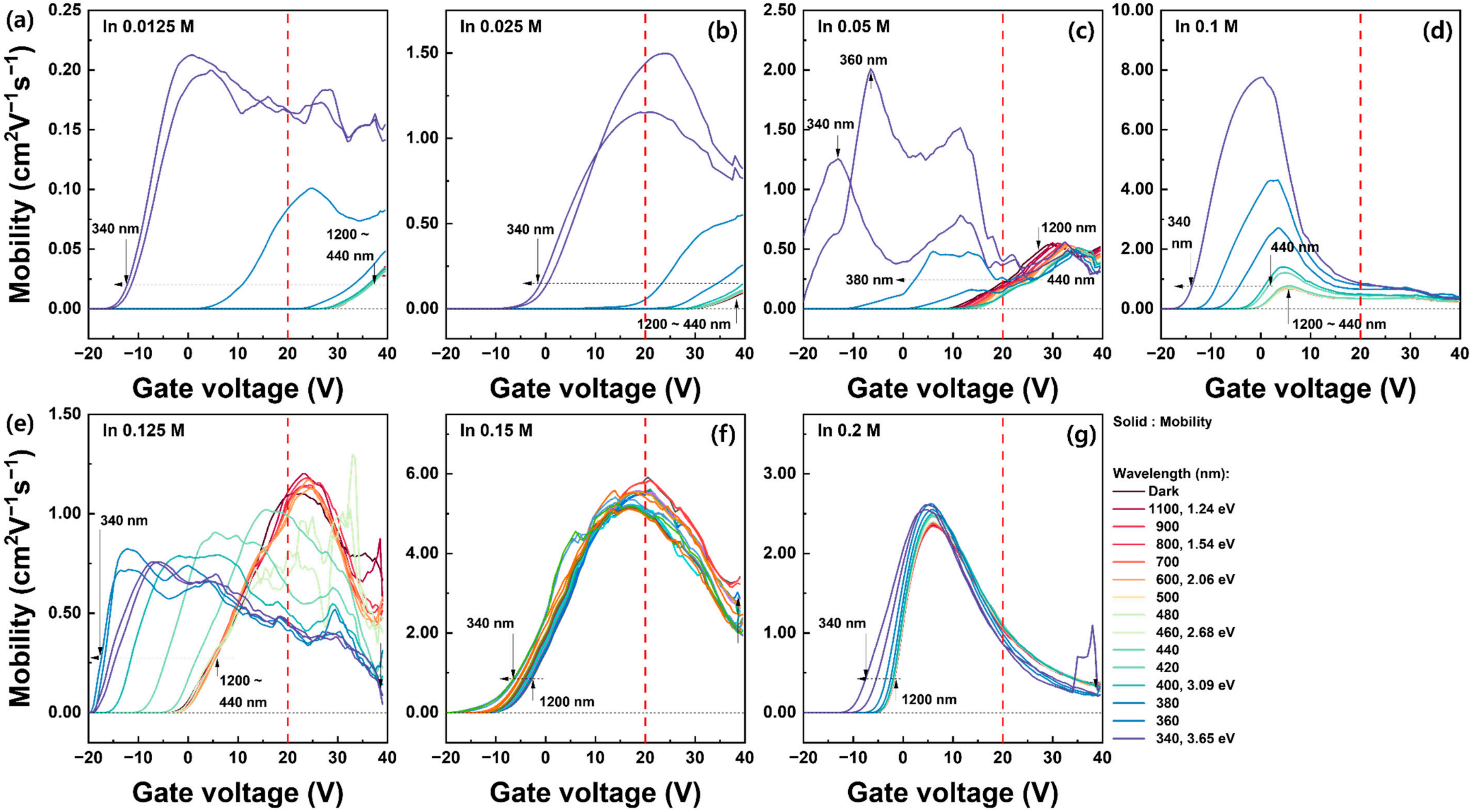

(a)
In 0.0125 M
340 nm
1200 ~ 440 nm
(b)
In 0.025 M
340 nm
1200 ~ 440 nm
(c)
In 0.05 M
360 nm
340 nm
1200 nm
380 nm
440 nm
(d)
In 0.1 M
340 nm
440 nm
1200 ~ 440 nm
(e)
In 0.125 M
340 nm
1200 ~ 440 nm
(f)
In 0.15 M
340 nm
1200 nm
(g)
In 0.2 M
340 nm
1200 nm
Mobility (cm^{2}V^{-1}s^{-1})
Gate voltage (V)
Solid : Mobility
Wavelength (nm):
Dark
1100, 1.24 eV
900
800, 1.54 eV
700
600, 2.06 eV
500
480
460, 2.68 eV
440
420
400, 3.09 eV
380
360
340, 3.65 eV

(Figure S7)
